# Unilateral GPi-DBS Improves Ipsilateral and Axial Motor Symptoms in Parkinson’s Disease as Evidenced by a Brain Perfusion Single Photon Emission Computed Tomography Study

**DOI:** 10.3389/fnhum.2022.888701

**Published:** 2022-05-11

**Authors:** Yuka Hayashi, Takayasu Mishima, Shinsuke Fujioka, Takashi Morishita, Tooru Inoue, Shigeki Nagamachi, Yoshio Tsuboi

**Affiliations:** ^1^Department of Neurology, Faculty of Medicine, Fukuoka University, Fukuoka, Japan; ^2^Department of Neurosurgery, Faculty of Medicine, Fukuoka University, Fukuoka, Japan; ^3^Department of Radiology, Faculty of Medicine, Fukuoka University, Fukuoka, Japan

**Keywords:** Parkinson’s disease (PD), unilateral, deep brain stimuation, globus pallidum internus (Gpi), subthalamic nucleus (STN), ipsilateral, axial, single photon emission computed tomography (SPECT)

## Abstract

**Introduction:**

Deep brain stimulation (DBS) is an effective treatment for advanced Parkinson’s disease (PD) with the targeting bilateral subthalamic nucleus or globus pallidus internus (STN or GPi-DBS). So far, detailed studies on the efficacy of unilateral STN-DBS for motor symptoms have been reported, but few studies have been conducted on unilateral GPi-DBS.

**Materials and Methods:**

Seventeen patients with Parkinson’s disease (PwPD) who underwent unilateral GPi-DBS were selected. We conducted comparison analyses between scores obtained 6–42 months pre- and postoperatively using the following measurement tools: the Movement Disorder Society Unified Parkinson’s Disease Rating Scale (MDS-UPDRS) part III, the Hoehn and Yahr stage, the presence/absence of dyskinesia, Mini-Mental State Examination (MMSE), Frontal Assessment Battery (FAB), Geriatric Depression Scale (GDS), levodopa equivalent dose (LED), and cerebral blood flow by single photon emission computed tomography (SPECT). Patient backgrounds were compared between four cohorts with favorable (good responders, ≥50% improvement) and unfavorable (poor responders, <50% improvement) postoperative outcome.

**Results:**

Significant improvement was observed postoperatively in the following: total MDS-UPDRS Part III scores during the off period, contralateral scores, ipsilateral scores, and axial scores. Similarly, the Hoehn and Yahr stages during the off period, and GDS also showed significant decrease. In contrast, LED, MMSE, and FAB remained unchanged while the number of patients who scored positive for dyskinesia decreased by 40%. Abnormal cerebral blood flow preoperatively seen in the cerebral cortex had normalized in the total score-based good responder cohort. In the ipsilateral score-based good responder cohort, cerebral blood flow increased in the contralateral frontal lobe including in the premotor cortex, contralateral to the DBS. Compared to the poor responders, postoperative good responders demonstrated significantly higher preoperative MMSE scores.

**Discussion:**

Unilateral GPi-DBS therapy was effective in improving contralateral, ipsilateral, and axial motor symptoms of patients with advanced PD; in particular, it was found to be especially beneficial in PwPD whose cognitive function was unimpaired; the treatment efficacy rivaled that of bilateral counterparts up till at least 6 months postoperatively. Finally, normalization of preoperative abnormalities in cerebral blood flow and increased cerebral blood flow in the contralateral frontal lobe indicated the beneficial potential of this therapy on ipsilateral motor symptoms.

## Introduction

Parkinson’s disease (PD) is a progressive neurodegenerative disorder characterized by both motor and non-motor symptoms such as tremor, muscle rigidity, bradykinesia, and inability to retain a suitable posture, in addition to dysosmia, dysautonomia, cognitive impairment, psychosis, and sleep disturbance. The effectiveness of dopamine replacement therapy (DRT) at the early stage of PD has been well-documented; DRT in combination with a routine exercise regimen enables long-term preservation of activity of daily living (ADL). Nevertheless, levodopa-induced motor complications, such as wearing off or dyskinesia, eventually emerge as the disease progresses and necessitate a dosage increase. Inevitably, ADL and quality of life (QOL) deteriorate as it becomes increasingly harder for patients to move freely (Berganzo et al., 2016; Gökçal et al., 2017). Upon reaching this stage, maintaining regular motor functions while controlling motor complications becomes an insurmountable challenge for patients with Parkinson’s disease (PwPD) even with the appropriate prescription of drug therapy and a routine exercise regimen. Fortunately, device-assisted therapies such as deep brain stimulation (DBS) and levodopa-carbidopa intestinal gel (LCIG) are available and have been shown to be highly effective for a carefully selected group of PwPD who reached such a plateau at advanced stages (Deep-Brain Stimulation for Parkinson’s Disease Study Group et al., 2001; Antonini et al., 2017).

By frequently stimulating two electrode targets implanted in the subthalamic nucleus (STN) and the internal segment of the globus pallidus (GPi), advanced PD symptoms improve under DBS therapy. Although differences in treatment effects do exist between STN and GPi, both therapies are equally useful in reducing both core motor symptoms and motor complications (Deep-Brain Stimulation for Parkinson’s Disease Study Group et al., 2001). While bilateral STN and GPi-DBS therapies have recently been established as standard treatment, there are also promising reports on the effectiveness of unilateral DBS therapies (Merello et al., 1999; Loher et al., 2002; Linazasoro et al., 2003; Germano et al., 2004; Chung et al., 2006; Kim et al., 2009; Okun et al., 2009, 2014; Walker et al., 2009; Zahodne et al., 2009). A steadily accumulating body of evidence (Kumar et al., 1999; Merello et al., 1999; Loher et al., 2002; Linazasoro et al., 2003; Germano et al., 2004; Chung et al., 2006; Nakamura et al., 2007; Kim et al., 2009; Okun et al., 2009, 2014; Walker et al., 2009; Zahodne et al., 2009) suggests that unilateral STN-DBS therapy improves motor symptoms even in research that evaluated motor symptoms separately from the contralateral, ipsilateral, and axial aspects (Kumar et al., 1999; Chung et al., 2006; Nakamura et al., 2007; Agostino et al., 2008; Tabbal et al., 2008; Walker et al., 2009; Hasegawa et al., 2020). Moreover, unilateral STN-DBS therapy is capable of improving ipsilateral symptoms in other movement disorders such as essential tremor (Peng-Chen et al., 2013). Other noteworthy benefits of unilateral STN-DBS therapy for PwPD include reducing dyskinesia, depression, and the levodopa equivalent dose (LED) (Linazasoro et al., 2003; Chung et al., 2006; Kim et al., 2009; Walker et al., 2009) as well as improving ADL and QOL (Kumar et al., 1999; Linazasoro et al., 2003; Chung et al., 2006; Walker et al., 2009; Zahodne et al., 2009; Okun et al., 2014). While there is plenty of evidence in the literature that supports the overall effectiveness of unilateral GPi-DBS on PD (Merello et al., 1999; Vingerhoets et al., 1999; Loher et al., 2002; Nakamura et al., 2007; Rodrigues et al., 2007; Okun et al., 2009, 2014; Zahodne et al., 2009), studies that examined the effect of unilateral GPi-DBS on motor symptoms of PD in the contralateral, ipsilateral, and axial sides are scarce (Merello et al., 1999; Loher et al., 2002). Thus, in order to clarify the effectiveness of unilateral GPi-DBS from a different angle and identify suitable patient candidates, this study conducted a detailed comparative analyses of the pre- and postoperative changes in motor and non-motor symptoms of PwPD who underwent unilateral GPi-DBS.

## Materials and Methods

**FIGURE 1:**
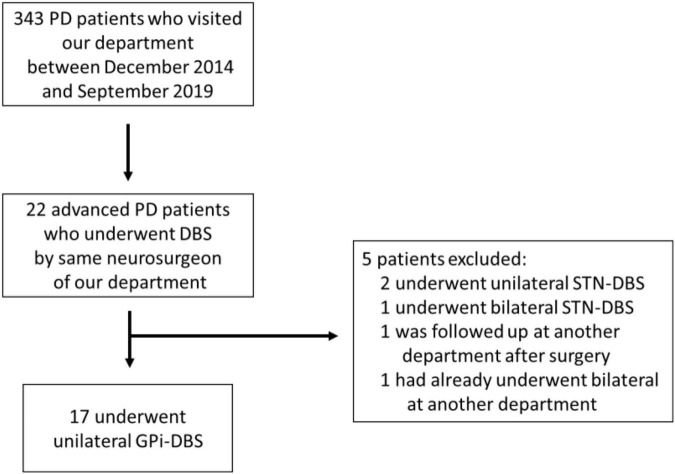
Flow chart of the study.

This is a retrospective observational study conducted at a single institution, the Fukuoka University Hospital. Of the database of 343 PwPD who visited our hospital during the period of 1 December 2014 to 30 September 2019, 17 patients, who received unilateral GPi-DBS stimulation to either right or left side and who were available for a 6 month postoperative assessment, were selected (Figure 1). All procedures were performed by a fellowship-trained functional neurosurgeon (T.M.), and the same DBS system was used in all study subjects (model 3387 DBS lead and Activa SC pulse generator, Medtronic, Minneapolis, MN, United States). Although our hospital is primarily focused on the staged GPi-DBS approach (Samii et al., 2007), we selected patients who requested either a unilateral or a staged DBS therapy when presented with the choice of bilateral, unilateral, or staged DBS therapy. These 17 patients all met the United Kingdom Parkinson’s Disease Society Brain Bank Clinical Diagnostic Criteria (UKBBC) and had been diagnosed with sporadic PD by a specialized neurologist (Hughes et al., 1992). Furthermore, all patients were preoperatively suffering from motor complications that: were difficult to control with a combined drug and exercise regimen; had shown >33% improvement in a total score obtained in the Movement Disorder Society Unified Parkinson’s Disease Rating Scale Part III (MDS-UPDRS Part III) in response to the levodopa challenge test (LDCT) that compared assessment during the off-stage and after taking levodopa; and were without prominent cognitive impairment [Mini-Mental State Examination (MMSE) score < 24] (Mishima et al., 2021). Concerning the programming, we follow the basic programming concept (Volkmann et al., 2002). Following testing the threshold levels of stimulation-induced side effects and therapeutic windows of monopolar stimulation at each contact, we usually select contact 1 or 2 as the active contact as the initial setting. Patients were instructed to regularly visit the programming clinic once a month for the stimulation adjustment performed by neurologists specializing movement disorders (Y.H., T.M., S.F., and Y.T.). The stimulation intensity and pulse width were gradually increased or decreased, and bipolar setting was selected when the intensity of monopolar stimulation reached the threshold level of side effects.

Measurements used for the assessment of motor and non-motor function respectively were as follows: MDS-UPDRS Part III, Hoehn and Yahr stages, presence/absence of dyskinesia; MMSE, Frontal Assessment Battery (FAB), and Geriatric Depression Scale (GDS). In addition, oral treatment was assessed with the levodopa equivalent dose (LED) (Tomlinson et al., 2010). A comparison analysis between pre- and approximately 6-month postoperative scores (mean, 6.6 ± 0.7 months; range, 6–7 months) was performed for each item. Both pre- and postoperative assessment with MDS-UPDRS Part III was undertaken during the off state. The assessment was conducted not only with total MDS-UPDRS Part III score, but also considered the ipsilateral and contralateral (total unilateral MDS-UPDRS Part III score from the sub-item 20–26) and axial scores (Kotagal et al., 2014) (total MDS-UPDRS Part III score from the sub-item 1, 9, 10, 12, 13) independently. In addition, two cohorts (Table 1) were created for comparison consisting of PwPD with favorable (good responder, total MDS-UPDRS Part III score ≥ 50%) and unfavorable (poor responder, total MDS-UPDRS Part III score < 50%) postoperative outcome. Comparisons were made in the following item categories obtained preoperatively: age, sex, disease duration, total MDS-UPDRS Part III score, Hoehn and Yahr stage (each on/off period), MMSE, FAB, GDS, LED, and presence/absence of dyskinesia.

**TABLE 1:** Comparison of each item at baseline between good responders (changes of Movement Disorder Society-Unified Parkinson’s Disease Rating Scale (MDS-UPDRS) part III total score after surgery ≧50%) and poor responders (<50%).

		Good responders (*n* = 9)	Poor responders (*n* = 8)	*P*
Age (years)		60.3 ± 5.5	64.4 ± 5.5	0.17
Sex (M:F)		4:5	3:5	0.77
Duration of PD (years)		9.9 ± 3.9	12.6 ± 6.1	0.61
LED (mg)		1,072.0 ± 363.8	990.6 ± 300.5	0.54
Hoehn and Yahr stage	Off	4 ± 0	4.1 ± 0.3	0.67
	On	2.7 ± 0.5	2.6 ± 0.5	0.89
MMSE		28.2 ± 0.8	26.9 ± 1.5	0.03
FAB		15.2 ± 1.2	15.8 ± 1.5	0.48
GDS		4.2 ± 2.8	4.8 ± 2.3	0.67
MDS-UPDRS part III total score		54.0 ± 12.4	48.6 ± 11.6	0.37
Dyskinesia (+: −)		7:2	8:0	0.77

**FIGURE 2:**
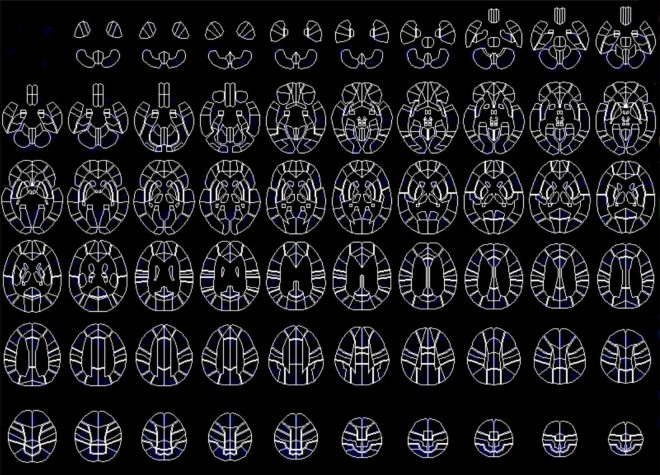
Fine stereotactic region of interest (ROI) template (SRT) image composed of 52 areas of ROI in Single Photon Emission Computed Tomography (SPECT) study.

Changes in pre- and approximately 11-month postoperative distribution of cerebral blood flow (mean, 11.1 ± 9.7 months; range, 6 months–3.5 years) were examined with ^99m^Tc-ECD single photon emission computed tomography (SPECT). SPECT data was missing from 3 patients (Case No. 4, 11, 17); therefore, the SPECT examination was based on available data from 14 patients. A SPECT examination was performed with patients’ eyes closed at resting state using a Technetium-99m ethyl cysteinate dimer (^99m^Tc-ECD) 600–900 MBq. A triple-detector gamma camera system (GCA-9300R; Cannon Medical Systems, Tokyo, Japan) was used for imaging. Data were collected during the period 5–16 min after a radioisotope (RI) was administered under the following conditions: 120°, 30 locations × 3, 120 s, a main energy window (20% of 141 KeV), a sub window (<7%). A high-resolution fan beam collimator was selected. SPECT images were corrected with 3D-OSEM reconstruction using absorptive correction (+), scatter−correction (+), μ value 0.15, and the Butterworth filter (BW); it was performed at order 4 (cut-off frequency, 0.13 cy/pixel), repetition time 10, and adding frequency of 10 times. The image analysis software eZIS (Fujifilm RI Pharma., Tokyo, Japan) was used to conduct image statistical analysis; this could be performed on a personal computer and was concordant with the patient SPECT image. Specifically, anatomically standardized SPECT images were compared with the images stored in the database of a standard brain of the corresponding age, for each patient’s data. Z-score for a region of interest (ROI) for each of 52 regions was determined with a fine stereotaxic region of interest template (Fine SRT) (Fuji Film, Tokyo, Japan) (Figure 2; Takeuchi, 2005). A total of four cohorts (Tables 2A,B) were created based on: (a) the total MDS-UPDRS Part III score, with favorable (good responder, ≥50%) and unfavorable (poor responder, <50%) postoperative outcome; (b) ipsilateral score, with favorable (good responder, improvement rate ≥ 50%) and unfavorable (poor responder, improvement rate < 50%) postoperative outcome (Antonini et al., 2003a). A comparison was made between pre- and postoperative Z-scores for each item category. For all item categories, paired *t*-tests were used for pre- and postoperative comparison and the Mann-Whitney test and the chi−square test were applied in the comparative analysis of good vs. poor postoperative responders. The threshold of statistical significance was established at *p* < 0.05 with a two-sided testing procedure. This study was approved by the Ethics Committee of the Fukuoka University Hospital (number, U20-04-001).

**TABLE 2A:** Comparison of *Z*-score between baseline and after unilateral deep brain stimulation of the globus pallidus internus (GPi-DBS) by brain perfusion Single Photon Emission Computed Tomography (SPECT).

Location*	Change in pre- and post-op CBF	*Z*-score		*p*	Normalization in post-op CBF
		Pre-op	Post-op		
**<Good responders>**					
Ipsilateral transverse temporal	↓	–0.304	0.154	0.03	Yes
Contralateral premotor	↑	0.344	0.026	0.03	Yes
Ipsilateral cingulate	↓	0.229	0.475	0.04	Not
Contralateral primary auditory**	↑	0.130	–0.049	0.04	Yes
Contralateral inferior parietal	↑	0.641	0.373	0.045	Yes
**<Poor responders>**					
Ipsilateral subcallosal	↑	–0.271	–0.842	0.01	Not
Ipsilateral inferior temporal	↓	–0.069	0.305	0.01	Not
Contralateral fusiform	↑	0.023	0.123	0.01	Not
Contralateral orbital	↑	–0.106	0.497	0.02	Not
Ipsilateral globus pallidus	↓	–0.212	0.426	0.02	Not
Ipsilateral substantia nigra	↓	–0.228	0.476	0.03	Not
Ipsilateral anterior cingulate	↓	–0.194	–0.456	0.03	Not
Contralateral paracentral lobule	↑	0.117	–0.166	0.03	Not
Ipsilateral orbital	↑	–0.251	–0.608	0.03	Not
Ipsilateral nucleus ruber	↓	0.021	0.575	0.03	Not

Patients were divided into good and poor responders in changes of Movement Disorder Society-Unified Parkinson’s Disease Rating Scale (MDS-UPDRS) part III total scores after surgery. *Only locations with significant changes in post-op CBF were extracted. **Primary auditory overlaps anatomically with frontal lobes. ↑: increased CBF, ↓: decreased CBF.

**TABLE 2B:** Comparison of *Z*-score between baseline and after unilateral deep brain stimulation of the globus pallidus internus (GPi-DBS) by brain perfusion single photon emission computed tomography (SPECT).

Location*	Change in pre- and post-op CBF	*Z*-score		*p*	Normalization in post-op CBF
		Pre-op	Post-op		
**<Good responders>**					
Ipsilateral transverse temporal	↓	–0.271	0.185	0.02	Yes
Contralateral premotor	↑	0.303	–0.001	0.02	Yes
Contralateral medial frontal	↑	–0.258	–0.482	0.02	Not
Contralateral inferior frontal	↑	–0.236	–0.569	0.03	Not
Contralateral Broca*	↑	–0.100	–0.443	0.03	Not
Contralateral Wernicke*	↑	0.676	0.301	0.03	Yes
Contralateral orbital	↑	–0.597	–0.908	0.04	Not
Contralateral middle frontal	↑	–0.091	–0.400	0.04	Not
Ipsilateral globus pallidus	↓	–0.476	0.318	0.04	Yes
Ipsilateral cingulate	↓	0.279	0.499	0.04	Not
Contralateral primary auditory**	↑	0.138	–0.021	0.04	Yes
Contralateral middle temporal	↑	0.104	–0.249	0.045	Not
**<Poor responders>**					
Ipsilateral parahippocampal	↓	–0.370	0.747	0.002	Not
Contralateral caudate head	↓	0.677	0.887	0.002	Not
Ipsilateral nucleus ruber	↓	–0.151	0.532	0.01	Not
Ipsilateral inferior temporal	↑	–0.079	–0.342	0.02	Not
Ipsilateral globus pallidus	↓	–0.243	0.261	0.02	Not
Contralateral fusiform	↓	–0.016	0.164	0.04	Not
Ipsilateral anterior cingulate	↓	–0.128	0.331	0.047	Not

Patients were divided into good and poor responders based on changes in Movement Disorder Society-Unified Parkinson’s Disease Rating Scale (MDS-UPDRS) part III ipsilateral sub scores after surgery. *These locations overlap anatomically with frontal and temporal lobes. **Primary auditory overlaps anatomically with frontal lobes. ↑: increased CBF, ↓: decreased CBF.

## Results

**FIGURE 3:**
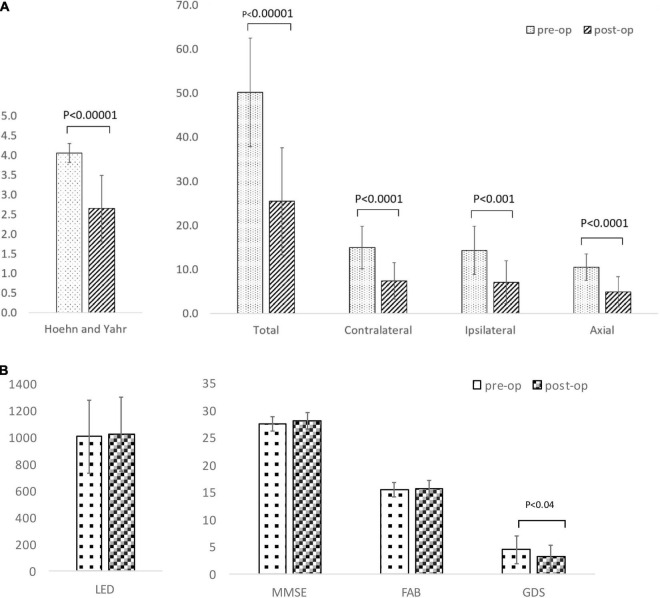
**(A)** Comparison of Movement Disorder Society-Unified Parkinson’s Disease Rating Scale (MDS-UPDRS) motor scores and Hoehn and Yahr stage between baseline and after unilateral deep brain stimulation of the globus pallidus internus (GPi-DBS). **(B)** Comparison of levodopa equivalent dose (LED), Mini-Mental State Examination (MMSE), Frontal Assessment Battery (FAB), Geriatric depression scale (GDS) between baseline and after unilateral deep brain stimulation of globus pallidus internus (GPi-DBS).

Table 3 gives the background data of the 17 patients who participated in our study: male to female sex ratio, 7:10; average age, 62.2 ± 5.8 years old; average disease duration, 11.2 ± 5.2 years, mean preoperative LED 1,033.7 ± 284.6 mg/day; average pre- and postoperative differences in Hoehn and Yahr stages during off period, 4.1 ± 0.2/2.8 ± 0.8. Only two patients met the EARLYSTIM criteria (Schuepbach et al., 2013). No adverse events such as intra- and postoperative bleeding or infection occurred during our study. All patients underwent CT postoperative day 9 to evaluate the electrode position in the GPi, and a board-certified neurosurgeon (T.M.) confirmed that there was no lead misplacement. Figures 3A,B show MDS-UPDRS Part III score, Hoehn and Yahr stage, MMSE, FAB, GDS, and LED. Compared to preoperative data, significant postoperative improvement was identified in the following item categories: total MDS-UPDRS Part III score, 50.2 ± 12.3 vs. 25.5 ± 12.2, improvement rate 50.7%, *p* < 0.0001; contralateral score, 15.0 ± 4.8 vs. 7.4 ± 4.1, improvement rate 48.1%, *p* < 0.0001; ipsilateral score, 14.4 ± 5.5 vs. 7.1 ± 4.9, improvement rate 50.6%, *p* < 0.0001; and axial score 10.5 ± 3.1 vs. 4.9 ± 3.4, improvement rate 53.6%, *p* < 0.0001. In all cases, postoperative total MDS-UPDRS part III score during the off period improved compared to preoperative (Supplementary Table 1). The Hoehn and Yahr stage for both on period (*p* < 0.0001) and off period (*p* < 0.04) were significantly decreased postoperatively. Although not significant, the number of patients with dyskinesia decreased from 15 (88.2%) to 9 (52.9%) after GPi-DBS therapy, while no changes were detected in LED. Similarly, MMSE and FAB were unchanged whereas GDS (4.5 ± 2.6 vs. 3.2 ± 2.1, *p* = 0.04) showed improvement. The postoperative good responder cohort (*n* = 9, including two patients who met the EARLYSTIM criteria) demonstrated significantly higher MMSE scores (28.2 ± 0.8 vs. 26.9 ± 1.5, *p* < 0.05) compared to postoperative poor responder counterparts even before undergoing GPi-DBS therapy (Table 1). No patient has displayed serious adverse events at the 6-month postoperative period, to date. Pre- and postoperative Z-scores for 14 patients who experienced ^99m^-Tc-ECD SPECT are presented in Table 2A (Comparisons were made between good and poor responders based on total MDS-UPDRS Part III score) and Table 2B (Comparisons were made between good and poor responders based on ipsilateral score). Two noteworthy changes were detected postoperatively. First, abnormalities in cerebral blood flow observed in the bilateral cerebral cortex before GPi-DBS had normalized postoperatively in the total MDS-UPDRS Part III score good responder cohort (*n* = 8, mean age 62.0 ± 3.2 years old, postoperative improvement rate 69.2 ± 14.5%). In contrast, the poor responder cohort (*n* = 6, 62.7 ± 4.3 years old 27.3 ± 8.3%) showed scores that further deviated from the normal value in the bilateral cerebral cortex postoperatively (Table 2A). The second noticeable change was the significant postoperative increase in blood flow in the contralateral frontal lobe of the ipsilateral good responder cohort (*n* = 9, 61.8 ± 3.2 years old, 74.0 ± 13.9%). However, scores for the bilateral cerebral cortex showed greater deviation from the normal value or baseline in the poor responder counterparts (*n* = 5, 63.0 ± 4.6 years old, 5.9 ± 36.0%) after GPi-DBS therapy (Table 2B).

**TABLE 3:** Baseline characteristics of 17 patients with advanced Parkinson’s disease received unilateral deep brain stimulation of the globus pallidus internus (GPi-DBS).

Case no.	Age (years), sex	Duration of PD preop (years)	LED (mg)	Dyskinesia	Hoehn and Yahr stage Off/on	MDS-UPDRS part III				MMSE	FAB	GDS
						Total	Contralateral	Ipsilateral	Axial			
1	58, M	5	1,035	−	4/2	62	14	23	11	28	16	0
2	60, M	15	1,483.7	+	4/2	56	13	17	12	28	17	0
3	67, F	10	1,348	+	4/2	35	16	7	6	30	13	6
4	74, M	25	719.7	+	4/3	48	16	14	11	28	15	2
5	65, F	8	700	+	4/3	65	17	20	15	28	15	7
6	67, F	7	875	+	4/3	74	22	21	18	26	17	6
7	62, M	6	1,098	+	4/2	64	21	17	14	29	15	4
8	66, F	19	1,404.7	+	4/2	50	16	14	10	25	16	1
9	61, F	5	537.05	+	4/3	41	11	11	11	27	15	6
10	68, F	14	1,098	+	4/3	62	14	23	11	30	15	5
11	65, M	12	1,300	+	5/2	42	11	9	11	27	15	6
12	59, M	9	1,100	−	4/3	37	14	9	8	28	15	3
13	54, M	11	925	+	4/3	41	15	13	5	26	17	3
14	65, F	5	1,024.1	+	4/3	46	14	11	9	27	14	4
15	58, F	14	660	+	4/3	64	18	21	10	28	13	6
16	61, F	12	815	+	4/3	58	16	22	10	26	18	8
17	48, F	13	1,449.95	+	4/3	30	8	5	8	28	17	9
Average ± SD	51.1 ± 6.5 (M:F) 7:10	11.2 ± 5.2	1,033.7 ± 284.6	(+: −)15:2	4.1 ± 0.2/ 2.65 ± 0.5	50.2 ± 12.3	15.4 ± 4.8	14.4 ± 5.5	10.5 ± 3.1	27.6 ± 1.3	15.5 ± 1.4	4.5 ± 2.6

## Discussion

At postoperative 6 months, motor symptoms of PwPD significantly improved in the axis and sides contralateral or ipsilateral to the target area treated with DBS. Given the 50.7% improvement rates of the total UPDRS part III scores obtained in this study, and the 24–67% (postoperative 6 months) (Kumar et al., 1999; Houeto et al., 2000; Molinuevo et al., 2000; Deep-Brain Stimulation for Parkinson’s Disease Study Group et al., 2001; Volkmann et al., 2001, 2009; Simuni et al., 2002; Thobois et al., 2002; Schupbach et al., 2005; Tanei et al., 2009a; Mei et al., 2020) or 38–56% (postoperative 3–7 months) (Vingerhoets et al., 1999; Deep-Brain Stimulation for Parkinson’s Disease Study Group et al., 2001; Volkmann et al., 2001; Loher et al., 2002; Tanei et al., 2009a) improvement rates from the bilateral STN or GPi-DBS in previous studies, it is reasonable to conclude that unilateral GPi-DBS therapy is equally as effective as bilateral DBS therapy. Improvement rates in our study (50.7%) were similar to the rates obtained in other unilateral GPi-DBS studies: 16.0–48.5% (Merello et al., 1999; Vingerhoets et al., 1999; Loher et al., 2002; Rodrigues et al., 2007; Okun et al., 2009, 2014; Zahodne et al., 2009). Contralateral scores also improved in similar rates to other studies (28.8–50.0%) (Merello et al., 1999; Loher et al., 2002) in the current study (48.1%). On the other hand, while improvement rates (23–24%) for the ipsilateral scores did not reach significance in other studies (Loher et al., 2002), our improvement rate was significant at 50.6%. There is only one study that previously showed a significant improvement in ipsilateral scores in unilateral GPi-DBS therapy; it evaluated the improvement solely on fingertip mobility (Nakamura et al., 2007). It is not clear what contributed to the remarkable improvement rates of the ipsilateral scores in this study; however, one might be the difference in measurement methods as MDS-UPDRS part III has not been used to assess motor symptoms of the ipsilateral side before this study. Interestingly, an equally remarkable improvement to ipsilateral scores was observed in the contralateral scores. Furthermore, improvement rate for axial score was notably high (56.7%) and rivaled improvements in contralateral and ipsilateral scores. This warrants further study since results concerning improvement rates for axial score after unilateral GPi-DBS therapy have been inconsistent: while Loher et al. (2002) reports a significant rate of improvement (41%), no improvement was identified in two other studies (Merello et al., 1999; Rodrigues et al., 2007). The current study assessed the significant differences between gait and postural stability scales of the MDS-UPDRS Part III; and freezing, walking, and balance scales in the Part II section of the same scale. The fact that we removed a total sum of item 1, 9, 10, 12, 13 (Takeuchi, 2005) in the MDS-UPDRS Part III of the motor examination section and defined them as an “axial score” may have contributed to the incomparably remarkable improvement in our study.

Studies (Merello et al., 1999; Loher et al., 2002; Volkmann et al., 2009) that assessed severity of dyskinesia after bilateral or unilateral GPi-DBS therapy, with the Rush Dyskinesia Rating Scale and the UPDRS part IV, have demonstrated a significant postoperative improvement. Although results were not significant, the number of patients who scored positive for dyskinesia (or who reported the presence of dyskinesia) during the on period decreased by 40%: from 15/17 preoperatively to 9/15 postoperatively. Caution must be exercised when interpreting this result; rather than the severity of dyskinesia, this analysis only focused on the presence or absence of dyskinesia.

After the unilateral GPi-DBS therapy, PwPD showed reduced depression; however, no change was detected in their cognitive function during the evaluation of non-motor symptoms. Consistent with other unilateral GPi-DBS studies (Loher et al., 2002; Simuni et al., 2002; Rothlind et al., 2007; Zahodne et al., 2009), this positive effect on depression found in the present study further increased confidence in GPi-DBS’s ability to ease depression in PwPD. Compared to the poor responder counterparts, the good responder cohort, who showed favorable outcomes in motor symptoms after unilateral GPi-DBS therapy, scored significantly higher in MMSE, indicating a better cognitive function. Thus, it is speculated that unilateral GPi-DBS is most effective for PwPD with preserved cognitive functions. Furthermore, the fact that the two cases that met the EARLYSTIM criteria in this study belonged to the good responder cohort, also suggests that the use of the unilateral GPi-DBS in the early stages of PD can be especially beneficial. Nevertheless, the small sample size warrants caution and further replication.

This study examined ^99m^ECD-SPECT pre- and postoperatively and noted that abnormal cerebral blood flow, preoperatively observed in the bilateral cortex, normalized after unilateral GPi-DBS in PwPD who showed improvement in motor symptoms. In addition, cerebral blood flow increased in the frontal lobe including in the premotor cortex contralateral to the side stimulated with DBS. The majority of studies that investigated correlations to improvement in motor symptoms with cerebral blood flow tomography or SPECT, concerned patients who underwent STN-DBS therapy. A potential link between the increase in cerebral blood flow in the motor-related areas of the frontal lobe (e.g., premotor cortex, pre-SMA, SMA, and anterior cingulate) and reduced motor symptoms after unilateral STN-DBS therapy has been suggested in ^99m^Tc-ECD SPECT studies (Sestini et al., 2002, 2005; Antonini et al., 2003a; Paschali et al., 2013). In addition, potential links between the postoperative normalization of abnormal blood flow (Cilia et al., 2009) in the bilateral cerebral cortex, nucleus basalis, or hypothalamic loop, and improvement in motor symptoms have been documented in the literature (Antonini et al., 2003b). Only a single assessment study (Tanei et al., 2009b) exists regarding the postoperative effect of unilateral STN-DBS; a significant vascular flow increase within the bilateral cingulate gyrus and cerebellum was identified, whereas vascular flow significantly decreased in both the bilateral medial frontal and superior temporal lobes. A significant correlation has also been found in the literature (van Laere et al., 2000) concerning GPi-DBS and ^99m^ECD-SPECT between decreased vascular flow in the ipsilateral thalamus and corpus striatum in relation to improvement in motor symptoms. However, comparison of the latter study to the current study is not relevant since the lesion effect of inserting electrodes serves as a confounding factor. Revitalized cortical activity was detected postoperatively in the motor-related areas of the frontal lobe, including in the ipsilateral side, in a unilateral GPi-DBS study using near-infrared spectroscopy (NIRS) (Morishita et al., 2016).

Insights are offered in the unilateral STN-DBS study, regarding the potential improvement mechanism in the ipsilateral symptoms. Motor symptoms of the ipsilateral side may be positively affected by the stimulation received by the ipsilateral pedunculopontine tegmental nucleus (PPN) (Nakano, 2000) transmitted through the contralateral side of the brain; this takes place via input from the ipsilateral supplementary motor area including the neuronal network of the cortex-basal ganglia-thalamus loop to the bilateral basal ganglia (Parent and Hazrati, 1995a,b; Chung et al., 2006; Tabbal et al., 2008) and input from the bilateral GPi and substantia nigra compacta into the bilateral thalamus and brain stem (Parent and Hazrati, 1995a,b; Levy et al., 1997; Chung et al., 2006; Tabbal et al., 2008; Peng-Chen et al., 2013). Based on our findings that showed a significant increase in cerebral blood flow in the contralateral frontal lobe, including in the premotor cortex, it is speculated that ipsilateral symptoms might be improved by using unilateral GPi-DBS therapy that stimulates the area contralateral to the side being stimulated in the cortex-basal ganglia-thalamus loop. The unilateral pyramidal tracts are involved in about 20% of control in motor functions of the body axis on the ipsilateral side (Germano et al., 2004). Thus, axial symptoms may be mitigated by improvement in blood flow of the unilateral premotor cortex areas. Previous study indicates that a deterioration in blood flow is noticeable in the frontal cingulate gyrus of PwPD who had dominant axial symptoms (Mito et al., 2006). Therefore, a significant increase in blood flow in areas that affect motor symptoms after unilateral GPi-DBS therapy, such as the frontal cingulate gyrus and frontal lobe areas including the premotor cortex, might contribute to the improvement in axial symptoms.

## Limitations of the Study

Despite being one of the few detailed studies on the effectiveness of unilateral GPi-DBS therapy, this study has several limitations. Firstly, readers should be reminded that this was a retrospective observational study based on data from a single institution and the period of observation only lasted for 6 months with a small sample size of 17 patients. Secondly, this study attempted to offer insights by comparing the analyses of patients who underwent unilateral GPi-DBS in the current study to similar previous studies. Because it is impossible to fully control the conditions of studies that have already been conducted, our interpretations may be confounded. Thirdly, the period when ^99m^Tc-ECD SPECT was performed postoperatively ranged widely; therefore, our assessment of cerebral blood flow may have been affected by other factors, such as the rate of progression in PD. Finally, this study examined the cerebral hemisphere ipsilateral or contralateral to the side where the DBS lead was inserted, without any clear knowledge of which side should be prioritized for which symptoms. In addition, this study did not evaluate the relationships between stimulation fields and the clinical responses/SPECT findings. Further studies are warranted to address these limitations.

## Conclusion

Regardless of these limitations, this 6-month postoperative assessment was valuable in that it underscored the potential of unilateral GPi-DBS therapy, in improving both motor- and non-motor symptoms including depression and in maintaining speech and cognitive function of PwPD, that does not pale in comparison to bilateral GPi-DBS therapy. The unilateral GPS-DBS therapy performed on PwPD, whose cognitive function remains unimpaired at relatively early stages of the disease, demonstrated therapeutic benefit equivalent to that of the bilateral counterparts, at over 6 months postoperatively. However, the results of this study constitute only a snapshot of information regarding the effect of unilateral GPi-DBS therapy and warrant a further investigation into long-term effects of the therapy and mechanisms responsible for mitigating various PD symptoms. Given the fact that unilateral GPi-DBS is less invasive and requires less battery energy than bilateral counterparts, the former could prove economically advantageous if it could systematically be shown that its effects on debilitating PD symptoms are long-lasting.

## Data Availability Statement

The raw data supporting the conclusions of this article will be made available by the authors, without undue reservation.

## Ethics Statement

The studies involving human participants were reviewed and approved by the University of Fukuoka Institutional Review Board. Written informed consent for participation was not required for this study in accordance with the national legislation and the institutional requirements.

## Author Contributions

YT, TMi, and YH: conception and design. YT, TMi, SN, and YH: analysis and interpretation of data. YH: drafting the manuscript. YT, TMi, SF, TI, TMo, and SN: revising manuscript critically for important intellectual content. YT: final approval of the version to be submitted. All authors contributed substantially to this study.

## Conflict of Interest

The authors declare that the research was conducted in the absence of any commercial or financial relationships that could be construed as a potential conflict of interest.

## Publisher’s Note

All claims expressed in this article are solely those of the authors and do not necessarily represent those of their affiliated organizations, or those of the publisher, the editors and the reviewers. Any product that may be evaluated in this article, or claim that may be made by its manufacturer, is not guaranteed or endorsed by the publisher.

## Abbreviations

ADL: activity of daily living
BW: Butterworth filter
DBS: deep brain stimulation
DRT: dopamine replacement therapy
Fine SRT: fine stereotaxic region of interest template
FAB: Frontal Assessment Battery
GDS: Geriatric Depression Scale
GPi: globus pallidus internus
LDCT: levodopa challenge test
LED: levodopa equivalent dose
LCIG: levodopa-carbidopa intestinal gel
MMSE: Mini-Mental State Examination
MDS-UPDRS: Movement Disorder Society Unified Parkinson’s Disease Rating Scale
NIRS: near-infrared spectroscopy
PD: Parkinson’s disease
PwPD: patients with Parkinson’s disease
PPN: pedunculopontine tegmental nucleus
QOL: quality of life
RI: radioisotope
ROI: region of interest
SPECT: single photon emission computed tomography
STN: subthalamic nucleus
STN-DBS: subthalamic nucleus deep brain stimulation
UKBBC: United Kingdom Parkinson’s Disease Society Brain Bank Clinical Diagnostic Criteria.
