# Supplementary material for: Unilateral GPi-DBS Improves Ipsilateral and Axial Motor Symptoms in Parkinson’s Disease as Evidenced by a Brain Perfusion Single Photon Emission Computed Tomography Study
Source: Front Hum Neurosci. 2022 May 11;16:888701. doi: 10.3389/fnhum.2022.888701 (PMC9130959; doi:10.3389/fnhum.2022.888701)
Supplement: Supplementary file 1 [file Table_1.DOCX]

Supplementary Table 1. Pre- and post-operative total Movement Disorder Society-Unified Parkinson’s Disease Rating Scale (MDS-UPDRS) part Ⅲ scores during the off period for each case are shown.

| Case No. | Pre-op | Post-op | Improvement rate (%) |
| --- | --- | --- | --- |
| 1 | 62 | 21 | 66.1 |
| 2 | 56 | 28 | 50.0 |
| 3 | 35 | 29 | 17.1 |
| 4 | 48 | 34 | 29.2 |
| 5 | 65 | 7 | 89.2 |
| 6 | 74 | 54 | 27.0 |
| 7 | 64 | 16 | 75.0 |
| 8 | 50 | 39 | 22.0 |
| 9 | 41 | 25 | 39.0 |
| 10 | 62 | 21 | 61.1 |
| 11 | 42 | 25 | 40.4 |
| 12 | 37 | 2 | 94.6 |
| 13 | 41 | 31 | 24.4 |
| 14 | 46 | 14 | 69.6 |
| 15 | 64 | 24 | 62.5 |
| 16 | 58 | 35 | 39.7 |
| 17 | 30 | 15 | 50.0 |
